# Deciphering the complex interplay of waterlogging and anthracnose twister disease in onion (*Allium cepa* L.)

**DOI:** 10.3389/fpls.2025.1580269

**Published:** 2025-09-19

**Authors:** Vanita N. Salunkhe, Sandeep B. Adavi, Suresh J. Gawande, Pratapsingh S. Khapte, Pranjali A. Gedam, Sushil S. Changan, K Sammi Reddy, Vijay Mahajan

**Affiliations:** ^1^ Indian Council of Agricultural Research (ICAR)-National Institute of Abiotic Stress Management, Baramati, Maharashtra, India; ^2^ ICAR-Directorate of Onion and Garlic Research, Pune, Maharashtra, India; ^3^ ICAR-National Institute of Biotic Stress Management, Raipur, Chhattisgarh, India

**Keywords:** post-infection waterlogging, pre-infection waterlogging, anthracnose, abiotic and biotic stress, cross tolerance

## Abstract

In the face of the escalating crisis of climate change and global warming, agricultural crops face unprecedented challenges with the dual threat of abiotic and biotic stresses. Onion (*Allium cepa* L.), a crop of therapeutic and culinary value, is particularly vulnerable. Among the three seasons cultivated in India, which ranks first globally in total area and production, the monsoon season (July to October) onion production in India is significantly challenged by waterlogging and anthracnose disease caused by *Colletotrichum gloeosporioides* species complex. The projected changes in the climate, including erratic rainfall, may amplify the severity of these challenges, and their co-occurrence may pose a significant threat to sustainable onion production. To investigate this interaction, a pot experiment was carried out with a focus on evaluating how waterlogging timing, prior to (pre-infection) and following infection (post-infection), influences the disease establishment and their cumulative effect on plant performance. To evaluate this, we exposed 90-day-old onion plants to pre- and post-infection waterlogging, along with separate individual stresses and controls, which were grown without any stress, and various morpho-physio-biochemical parameters were recorded at two intervals (7 days after the first and secondary stress). We observed that plants receiving only an inoculum spray showed significantly higher plant height with enhanced pseudostem (51 cm), while waterlogging hindered it, with pre-infection waterlogging showing a 19% reduction. The post-infection waterlogging significantly suppressed the plant growth, reduced the chlorophyll and carotenoid content (nearly threefold), elevated the membrane injury, and displayed a notable decline in the relative water content (RWC) and protein content (69%–73%) compared to either individual or pre-infection waterlogging. Additionally, the post-infection waterlogging elicited a stronger antioxidant activity (SOD and APX), indicating more robust oxidative response. In contrast, the pre-infection waterlogging seemed to partially inhibit the pathogen establishment primarily due to hypoxia-induced plant defense response. These findings highlight the timing of the stress occurrence, with post-infection waterlogging exacerbating the disease progression, severity, and damage and pre-infection waterlogging offering partial tolerance. Understanding such a dynamic interaction will help in developing integrated management as well as developing a resilience onion cultivar to both biotic and abiotic stress through breeding strategies.

## Introduction

Over the years, climate change has progressively increased the frequency of extreme weather events and altered the availability of water, which significantly affects the growth and survival of plants (IPCC, W, 2021; Rezaei et al., 2023). Waterlogging, the second most important abiotic stress after drought, is shown to affect 16% of the global cultivated area. Between 2008 and 2018, the occurrence of persistent floods and waterlogging has severely affected crop and livestock production, causing economic loss of US$49 billion in Asia (FAO, 2021). Roots are the foremost organ influenced by waterlogging, in which soil aeration is compromised, leading to a hypoxic or anoxic condition that elicits anaerobic fermentation, depleting the overall plant energy level and consequently inhibiting root growth. Additionally, the limited oxygen level alters the soil microbiome along with soil physicochemical properties like redox potential and pH. Furthermore, waterlogging curtails the accumulation of oxidized nutrient elements (NO_3_^-^ SO_4_^2−^, Fe^3+^) while enhancing the accumulation of reduced elements (NH_4_^+^, Fe^2+^, Mn^2+^, H_2_S), which are toxic to plants along with carbon dioxide and ethylene which are synthesized internally (Greenway et al., 2006; Kreuzwieser and Rennenberg, 2014). These altogether result in oxidative bursts and photo-oxidative damage, which stem a cascade of events, damaging the cell membrane, deactivating enzymes, disrupting the translocation of water and nutrients, and vitiating photosynthesis (Herzog et al., 2016). The impaired photosynthesis under waterlogging arises from stomatal and non-stomatal limitations. The stomatal limitations are indicated by limited stomatal conductance due to the lower hydraulic conductivity of the root, which impairs nutrient and water absorption, and non-stomatal limitations include alteration in the activity of RuBisCO and PEPCase enzymes, reduction in chlorophyll pigment, and accumulation of carbohydrate (Iglesias et al., 2002). These physiological changes will significantly affect the yield, which was evident from earlier studies on onion, okra, and maize (Bange et al., 2004; Vwioko et al., 2017; Gedam et al., 2022).

Plants have evolved multiple mechanisms to tolerate waterlogging stress. Morphological adaptations often include the development of aerenchyma, adventitious roots, and lenticels as well as features such as radial oxygen loss (ROL) barriers, cuticular air films, and rapid elongation of apical meristems to maintain oxygen supply under hypoxic conditions (Jackson, 2002; Bailey-Serres et al., 2012; Yohan et al., 2017; Liu et al., 2020). These responses are well documented in model crops like rice and mungbean, where changes in hormonal signaling and metabolic adjustments are known to support survival (Yang and Hoffman, 1984; Islam et al., 2022; Adak et al., 2021). At the metabolic level, hypoxia often enhances the activities of pyruvate decarboxylase (PDC) and alcohol dehydrogenase (ADH), favoring anaerobic respiration (Pan et al., 2021), and increases sucrose degradation through upregulated sucrose synthase (SuSy) activity (Kuai et al., 2016). Additionally, reactive oxygen species (ROS) generated under waterlogging can disrupt chloroplast ultrastructure, causing thylakoid disorganization, reduced chlorophyll fluorescence, and impaired photosynthesis (Zhou et al., 2020). Mitochondrial dysfunction and elongation further suppress respiration, leading to lipid peroxidation and damage to membranes, proteins, and nucleic acids, ultimately causing cell death (Ren et al., 2016; Wang et al., 2022). Whether onions exhibit similar anatomical or hormonal adaptations is unclear and remains hypothetical. Therefore, the present study focused on morphological and physiological traits directly observed in onion rather than on mechanisms inferred from other crops.

Onion (*Allium cepa* L.) is a vegetable crop whose significance in international trade arises due to its culinary and therapeutic value (Salunkhe et al., 2022). Its nutritional value arises from high levels of vitamins (β-carotenes and vitamin C), proteins, minerals like iron, calcium, sulphur, sugar compounds like arabinose, galactose, fructose, and glucose, and certain flavonoids and polyphenols. Traditionally, onion is used to cure minor injuries, gastrointestinal problems, worms, and microbial growth and to treat skin diseases (Kandoliya et al., 2015). Though India is the largest producer of onion (100.9 million tons) (FAO, 2023), it has lower productivity, lagging behind nations like South Korea, USA, and Australia (19.13 t/ha). This low productivity arises due to various biotic and abiotic stresses, including waterlogging and anthracnose (Gedam et al., 2022; Khar et al., 2022; Salunkhe et al., 2022). The shallow rooting system of the onion makes it more vulnerable to waterlogging, resulting in yield loss of 50%–70% when it occurs at the bulb development stage (Drinkwater and Janes, 1955; Ghodke et al., 2018). Among the three cropping seasons of onion in India, the monsoon season (July to October) onion is shown to be highly vulnerable to heavy rainfall and waterlogging, which significantly destabilizes the onion market during the months of October to January. The effect of waterlogging is further determined by various factors, viz., season, type of soil, rainfall extent, crop variety, and crop growth stages (Salunkhe et al., 2022).

These changes in the plant system in response to waterlogging, along with the congenial microclimate created, will facilitate the establishment of various diseases with increased susceptibility, causing a significant yield loss (Hsu and Shih, 2013; Savary et al., 2019; Sorrenti et al., 2019). Research has demonstrated that when oomycetes such as *Phytophthora*, *Pythium*, and *Phytopythium* or fungal diseases like *Fusarium* are present, flooding or waterlogging accelerates the course of the disease (Wilcox and Mircetich, 1985; Moslemi et al., 2018). These also seriously harm waterlogged plants, which leads to a high death rate. In the case of onion, anthracnose or twister disease caused by *Colletotrichum* spp. is of significant concern in the region where onion is cultivated in the rainy season and which is usually exposed to high rainfall and waterlogging (Baysal-Gurel et al., 2014; and Kim et al., 2008; Rodriguez-Salamanca et al., 2012; Salunkhe et al., 2022; Santana et al., 2016; Wiyono, 2007). Substantial work has revealed the possible involvement of *Fusarium* spp. in the development and augmentation of the disease symptom severity (Weeraratne, 1997; Kuruppu, 1999; Alberto and Aquino, 2010; Dutta et al., 2022). Waterlogged conditions often create a favorable environment for opportunistic soil-borne pathogens such as *Fusarium* spp. These pathogens can infect stressed plants through roots or wounds, leading to systemic infections or weakening the plant’s overall defense mechanisms, weakening plant tissues and making them more susceptible to *Colletotrichum* spp. Thus, *Fusarium* infection under waterlogged conditions could act synergistically with anthracnose, leading to more severe lesions and a faster decline in plant health. Studies on tomatoes and peppers have demonstrated that overhead irrigation and rainfall facilitate the dispersal of conidia of *Colletotrichum* spp. and also increases leaf wetness, creating optimal conditions for disease establishment and progression (Dillard, 1992; Sanogo et al., 2008). Although previous studies have explored the effects of waterlogging (Alberto et al., 2019; Dubey et al., 2021) and anthracnose (Gedam et al., 2022; Ghodke et al., 2018) on onion independently, they have largely overlooked the potential interactions between these stresses. Most existing research examine individual stress factors under controlled conditions. Consequently, the combined impact of waterlogging and anthracnose on onion growth, disease dynamics, and productivity remains poorly understood. This critical gap underscores the need for studies that address the interactive effects of multiple stresses to develop effective management strategies to minimize yield losses and to curtail price volatility by breeding crop varieties with resistance to combined stresses. Keeping this in mind, the present study aimed to assess the impact of waterlogging and anthracnose on morpho-physio-biochemical changes in onion plants and to determine whether pre-infection or post-infection waterlogging has a more severe effect on plant growth and development.

## Materials and methods

The present study was conducted in a screen house facility at ICAR —National Institute of Abiotic Stress Management (NIASM), Malegaon, Baramati, Maharashtra, India (18° 09′ 30.62″ N, 74° 30′ 03.08″ E) during the Kharif/monsoon season of 2023 (June to October). The study’s main aim is to understand the combined effect of waterlogging and anthracnose in two onion cultivars.

### Plant material and growing conditions

Based on our preliminary study with anthracnose disease (data not presented), two onion cultivars were selected for this study, Bhima super (*Kharif*, moderately resistant) and Bhima shakti (*Late Kharif/Rabi*, highly susceptible), both of which were obtained from ICAR —Directorate of Onion and Garlic Research (DOGR), Rajgurunagar, Pune and were grown on a raised nursery bed for 45 days. After 45 days, the seedlings were transplanted into 20- cm-diameter plastic pots containing a 3:1 ratio of soil and FYM in a screen house. The recommended package of practices comprising cultural practices, fertilizer application, and weed–insect–pest and disease management was followed to obtain a healthy plant (Gedam et al., 2022). The environmental parameter during the whole experiment is given in **Supplementary Table S1**. Five seedlings were maintained in each pot. After 45 days of transplanting, the treatments were applied as earlier research has shown that waterlogging at the bulb initiation and development stage causes 50%–70% yield reduction (Gedam et al., 2022). The experiment consists of five treatment combinations which are as follows: T1 — control (normal growing condition), T2 — waterlogging (10 days) stress, T3 — anthracnose (*Colletotrichum gloeosporiodes*) disease stress, T4 —post-infection waterlogging (initial 10 days anthracnose infection followed by 10 days of waterlogging), and T5 —pre-infestation waterlogging (initial 10 days of waterlogging followed by 10 days of disease infection). The 10 days of waterlogging were followed as per an earlier study done in ICAR-DOGR (Gedam et al., 2022). Each treatment consisted of four pots, with each pot carrying five seedlings. The waterlogging treatment was given by dipping the pots in plastic tanks and by maintaining the water level up to 5 cm of the pseudo stem with consistent replenishment of water (Gedam et al., 2022).

**Figure 1:**
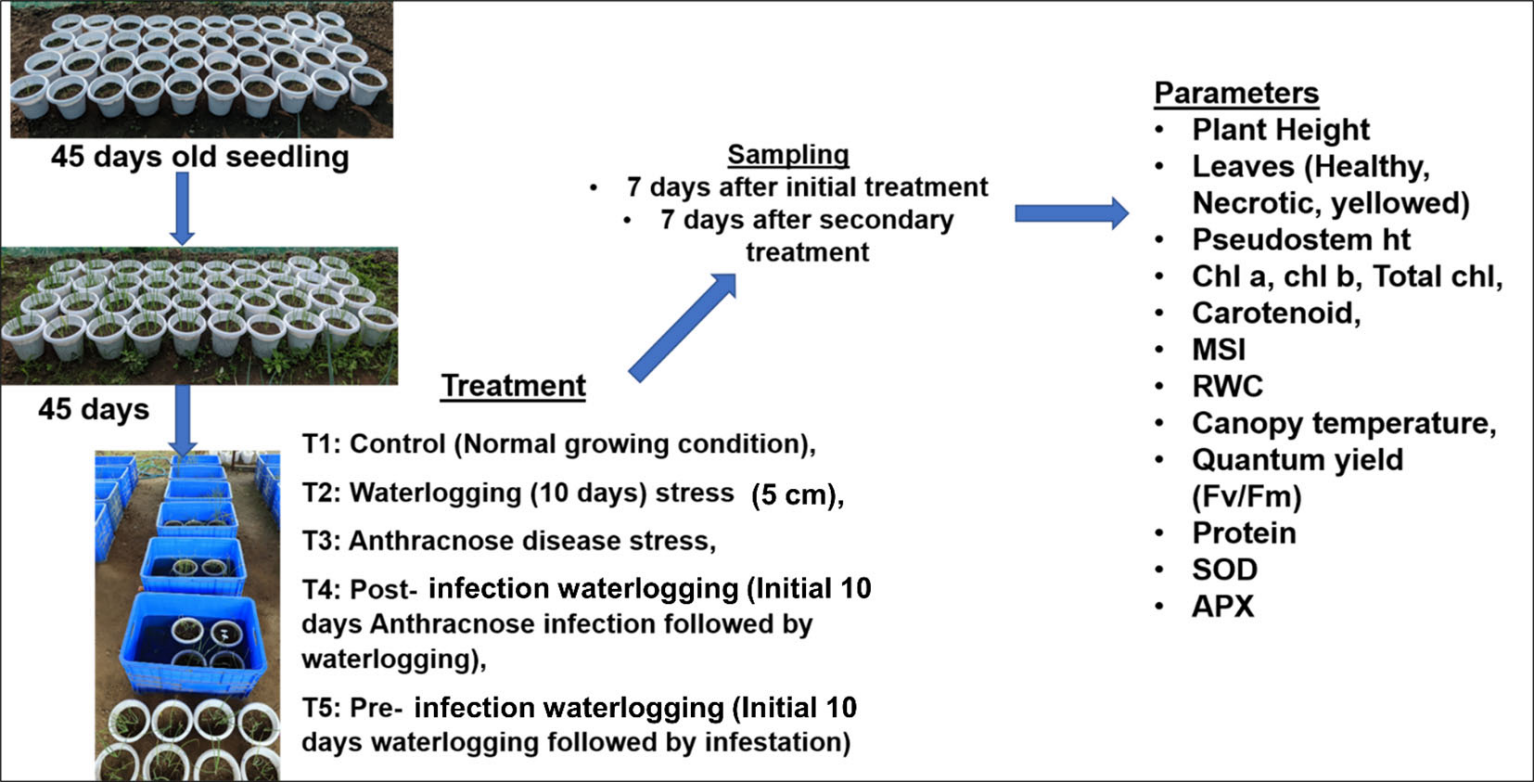
Experimental setup of the present study. The onion seedlings were grown for 45 days in a raised nursery bed and later transferred to pots and were grown for another 45 days. The 90 days old plants were exposed to various treatments as mentioned above and the various morpho-physiological and biochemical parameters were estimated as mentioned above.

The anthracnose-infected onion plant leaves collected from the ICAR-NIASM research field were used for the isolation of the pathogen. Primarily, the infected leaves were surface-sterilized with 2% sodium hypochlorite (NaOCl), sectioned into small pieces, and placed on oatmeal agar amended with streptomycin sulphate (3 mg/L) in petri plates. The culture plates were incubated at 25 °C ± 2 °C for 48 – 72 h. The fungus was identified based on morphological and cultural features (Weir et al., 2012). A highly sporulating and profuse growth strain of *C. gloeosporioides* was chosen for the screening experiment out of all the isolates. The chosen isolate’s spore suspension was made by rubbing and cleaning 7-day-old culture plates with double-distilled autoclaved water. The suspension was then filtered through a double layer of cheesecloth and diluted using a hemocytometer to 1 × 10–^6^ spores/mL. The anthracnose inoculation was done by spraying *C. gloeosporiodes* spores at 1×10^6^ spores/mL. The control plants were sprayed with sterilized water. The pathogen-inoculated pots were covered with a polythene bag to create a congenial environment for pathogen incubation, and the polythene bags were removed after 48 h. Regular water spraying was done both in the morning and evening hours to maintain favorable conditions for disease establishment. The morpho-physiological and biochemical parameters were recorded after 7 days of each treatment, i.e., first measurement after 7 days of initial stress and later second measurement after 7 days of subsequent stress. The experimental setup is depicted in **Figure 1**, and the weather data of the experimental site is given in **Supplementary Table S1**.

### Measurement of morphological parameters

The plants’ height was measured using a ruler for each genotype at two intervals as mentioned earlier. The observations were presented as the height per plant in centimeters (cm). Similarly, the pseudo stem length was measured using a ruler from the soil level until the plants displayed leaves and expressed in centimeters per plant. The observations were recorded in four replications for each treatment. The total number of leaves present in plants was counted manually from each plant in four pots. Along with this, the number of necrotic leaves and yellowed leaves was also counted. From these data, the percentage of healthy leaves and necrotic and yellow leaves was determined.

### Measurement of canopy temperature/shoot thermography

To obtain the thermal images of the shoot, we followed the method described earlier (Bayoumi et al., 2014). The thermal image of the plant canopy was obtained in the morning hours between 10 a.m. and 11 a.m. The images were obtained from approximately 1- m distance. The obtained thermal images in IRB format were converted into Fluke thermal images using SmartView 4.3TM Researcher Pro (Fluke Thermography, Plymouth, MN, USA). The images were taken from all four pots, and the temperature was noted by selecting multiple points from the each plant in such a way that covers all the possible temperature ranges from the leaf (Naguib et al., 2021). The temperature was averaged from each pot to ensure measurement accuracy.

### Estimation of photosynthetic pigments and chlorophyll fluorescence

The photosynthetic pigments were quantified by following the method described by Hiscox and Israelstam (1979). The second lower leaves were collected from the plant from each pot, and freshly harvested leaf samples were cut into small pieces measuring approximately 2 mm in length. The 20-mg leaf sample was dipped in 5 mL of dimethyl sulphoxide (DMSO) in a test tube and kept at 65° C for 30 min. Subsequently, they were kept overnight in the dark to drain out all the photosynthetic pigments into the DMSO solution. The absorbance was measured at 470, 645, and 663 nm in Thermo Scientific Multiskan GO, a high-quality monochromator-based UV/VIS spectrophotometer. The calculation for determining chlorophyll *a*, chlorophyll *b*, total chlorophyll, and total carotenoids was done according to Arnon (1949) and expressed as milligrams per gram of dry weight (mg g^-1^ DW). Furthermore, the ratio of chlorophyll *a*/*b* as well as total chlorophyll/total carotenoids was also calculated.Damage caused by the combined and individual stress to the photosynthetic system was estimated by measuring the quantum yield of PS II. The freshly harvested leaves from the plants were kept in dark condition for 30 min, and later the chlorophyll fluorescence was measured and the quantum yield (Fv/Fm) was estimated using FluorCam v. 7.0 software following the methodology described earlier (Krause and Weis, 1991; Schreiber et al., 1995; Basavaraj et al., 2024).

### Estimation of relative water content, membrane stability index, and membrane injury index

To estimate the relative water content (RWC), the leaf of the representative plant was taken, and the fresh weight was recorded. Later, the leaf sample of known weight was completely immersed in double-distilled water for 4 h until it attained full turgidity. Following 4 h of incubation, the turgid weight of the sample was recorded after gently removing the surface-adhered water using a tissue paper. Later, the sample was kept for drying in an oven at 60 °C until it attained a stable weight, and then the dry weight was recorded. The RWC was calculated using the formula given by Weatherley (1950).

The membrane stability index (MSI) was determined by estimating the ion leakage at two different temperatures, for which we followed the method described by Sairam et al. (1997). To maintain homogeneity, the second lower leaves were collected from each plant from each pot. The leaf samples were cut into small pieces of 2 mm, and the homogenized mixture of leaves weighing 200 mg was taken and placed in a test tube containing 20 mL of double-distilled water. The tubes were incubated at 40° C for 30 min in a water bath, and subsequently, the electrical conductivity (EC) was measured and noted as C1. Furthermore, the same test tubes with the sample were incubated in a water bath at 100°C for 10 min. Once the solution cooled down to room temperature, the EC was measured and noted as C2. The MSI was estimated by using the formula MSI = (1 - (C1/C2)) * 100. The estimated MSI is expressed in percentage (%). Furthermore, membrane injury (MI) is estimated by using the formula MI = 100 - MSI and expressed in terms of percentage (%).

### Estimation of protein content and activity of superoxide dismutase and ascorbate peroxidase

Enzyme activities were expressed per milligram of protein after determining the soluble protein content. For estimation of protein, 2 mL of Coomassie Brilliant Blue G - 250 was mixed with an appropriate quantity of plant extract and vortexed. The mixture was incubated at room temperature for 2 min, and absorbance was measured at 595 nm in Thermo Scientific Multiskan GO, a high-quality monochromator-based UV/VIS spectrophotometer (Bradford, 1976).

The activity of SOD was estimated as described by Dhindsa et al. (1981), wherein the reduction in formazone optical density was estimated, which was formed by a reaction between nitro-blue tetrazolium (NBT) dye and superoxide radical. Then, 1 g of freshly harvested leaf samples was ground in phosphate buffer (0.1 M; pH 7.8) comprising EDTA (0.5 mM) and ascorbic acid (1 mM). The filtrate obtained after passing the ground sample mixture through a cheesecloth was centrifuged for 20 min at 15,000 *g*. The supernatant obtained was further used for the estimation of enzyme activity. The enzyme assay mixture consists of EDTA (0.1 mM), NBT (75 µM), sodium carbonate (50 mM), methionine (13.33 mM), phosphate buffer (50 mM; pH 7.8), and enzyme solution (0.05 to 0.1 mL), and the final volume was adjusted to 3 mL. The reaction was instigated by adding 2 mM riboflavin, which was subsequently exposed to 15 min of light, and later the absorbance was measured at 560 nm. The activity of SOD was expressed as units which represent the quantity of enzymes required to attain a 50% absorbance reduction in comparison to the control.

The activity of ascorbate peroxidase (APX) was estimated as illustrated by Nakano and Asada (1981), where in the reduction in absorbance due to ascorbic acid consumption is measured at 290 nm. The reaction mixture comprised ascorbic acid (0.5 mM), EDTA (0.1 mM), potassium phosphate buffer (50 mM, pH 7.0), hydrogen peroxide (H_2_O_2_, 3 mM), and enzyme extract (0.1 mL), and the total volume was made to 3 mL with double-distilled water. The reaction was instigated with the addition of 0.2 mL H_2_O_2,_ and subsequently, absorbance reduction was noted for 30 s in a UV-visible spectrophotometer at 290 nm.

### Statistical analysis

The present investigation was executed with two onion cultivars and with five treatments in a pot culture experiment with a two-factor factorial CRD, with cultivar and treatment as the two factors. The sampling was done at two stages and with three replications for each measurement. R program was used to compute a two-way analysis of variance (ANOVA) with genotype, control, and infection treatment effects to compute adjusted *P*-values and the level of significance. Mean separation was done using Duncan Multiple-Range Test (DMRT) comparisons test “ visvaR” R package (Ramasamy, 2024). Graph Pad Prism version 9.0.0 (La Jolla, CA, USA) was used to prepare the graphs.

## Result

### Changes in plant morphological features

**Figure 2:**
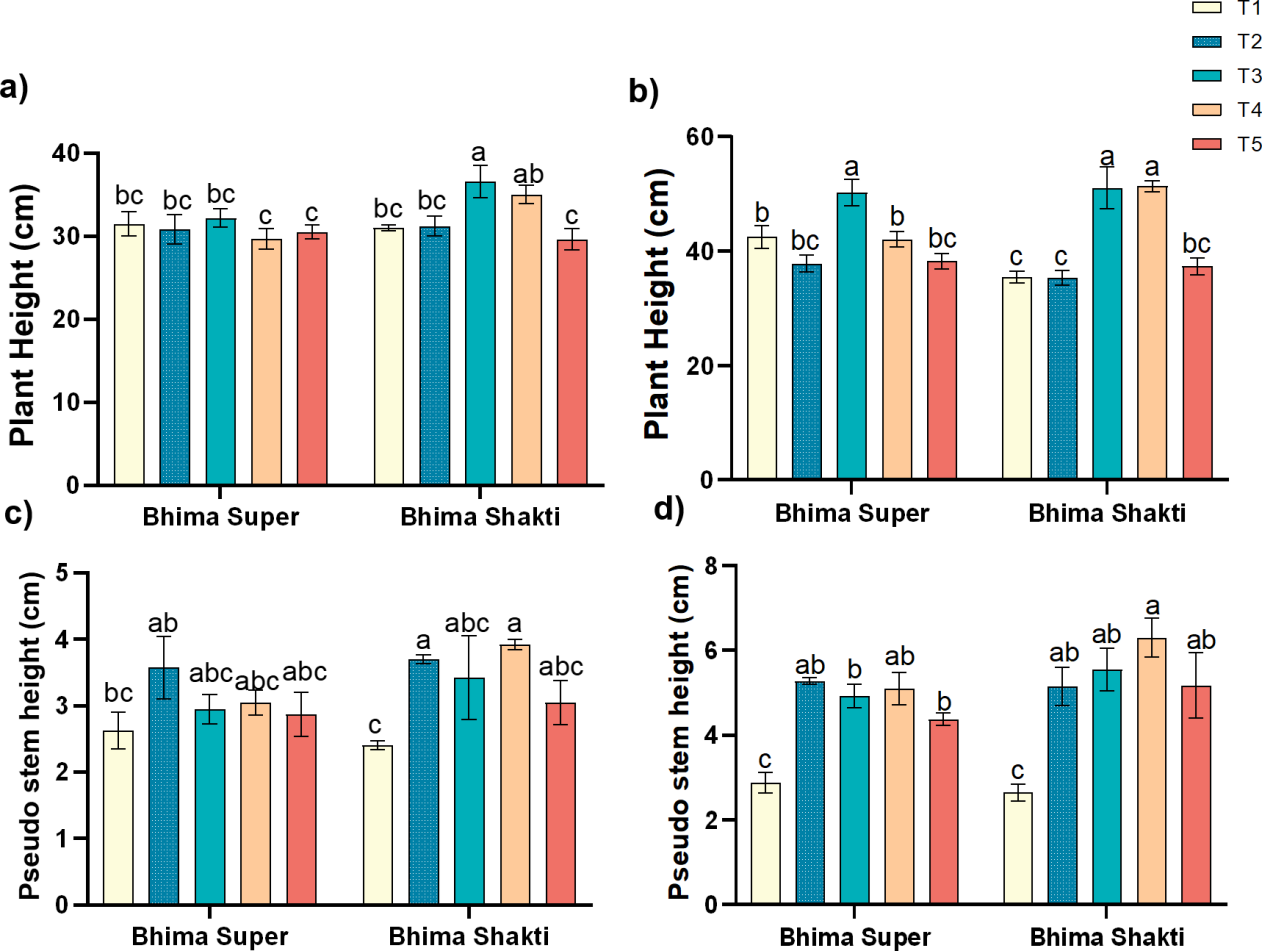
Effect of waterlogging and anthracnose on plant height **(a, b)** and pseudostem height **(c, d)** of Bhima Super and Bhima Shakti after 7 days of initial treatment **(a, c)** and 7 days of secondary treatment **(b, d)**. T1: Control, T2: Waterlogging, T3: Anthracnose, T4: Post- infection waterlogging, T5: Pre-infection waterlogging. The mean from the three biological replicates is represented along with standard error and Duncan’s multiple comparison for mean separation.

Pre-infection and post-infection waterlogging significantly affected the plant morphological features comprising plant height, pseudostem length, and damage caused to the leaves in both cultivars. The height of onion plants varied between 29.65 and 36.62 cm across cultivars and treatments at 7 days after the initial treatment. The cultivar Bhima Super did not show any significant changes in plant height at 7 days after treatment, while in Bhima Shakti the anthracnose infection showed approximately 11%–15% increase in height than the control plants. At 7 days after the second treatment, the height of the onion plant was highest in plants receiving sole anthracnose treatment, i.e., approximately 51 cm, while the lowest was seen in plants receiving waterlogging treatment. In the case of Bhima Super, pre-infection waterlogging treatment was shown to reduce the plant height by ~19% in comparison to sole inoculum-treated plants. On the other hand, the plant height in post-infection waterlogging did not show any significant changes in comparison to the sole waterlogged condition. These indicate that the increase in plant height is mainly due to the sole effect of the pathogen while waterlogging has suppressed the plant height (**Figures 2a, b**). The pseudostem height ranged between 2.6 cm and 3.6 cm across treatments in Bhima Super, while in Bhima Shakti, it ranged between 2.4 cm and 3.9 cm after 7 days of the initial treatment. Post-infection waterlogging treatment has shown a similar level of pseudostem elongation to that of sole inoculum spray treatment, while pre-infection waterlogging has exhibited a comparatively lower elongation of pseudostem across cultivars. This indicates that waterlogging and disease both play a significant role in pseudostem elongation. In Bhima Super, the pseudostem elongation was 60%–82% higher than the control, while in Bhima Shakti the enhancement was 90%–130% (**Figures 2c, d**).

**Figure 3:**
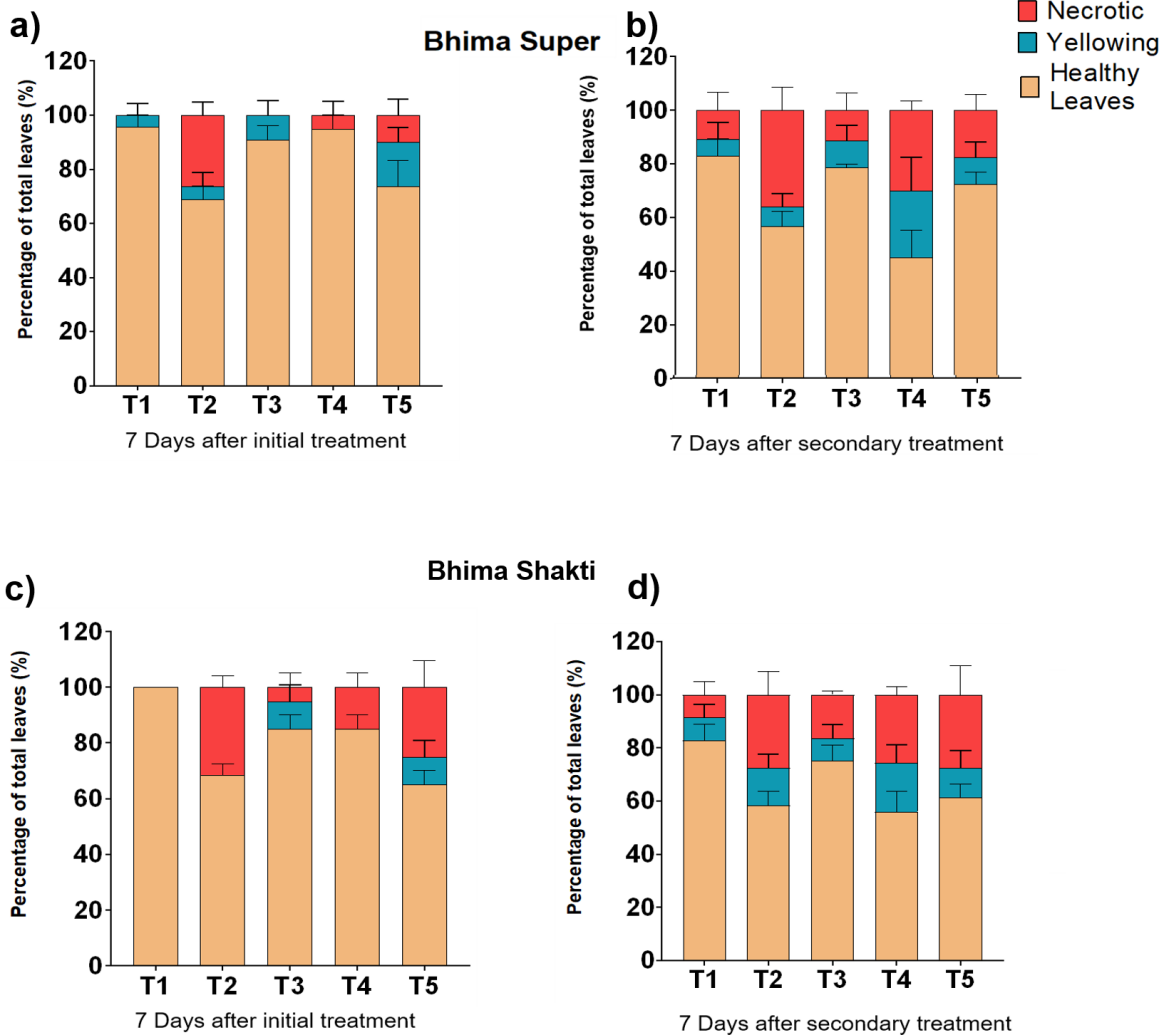
The percentage of leaf damage caused by waterlogging and anthracnose in Bhima Super **(a, b)** and Bhima Shakti **(c, d)** after 7 days of initial treatment **(a, c)** and 7 days of secondary treatment **(b, d)**. T1: Control, T2: Waterlogging, T3: Anthracnose, T4: Post- infection waterlogging, T5: Pre-infection waterlogging. The mean from the three biological replicates is represented along with standard error.

The total number of leaves varied between four and seven across cultivars. After 7 days of waterlogging, around 26%–30% of the leaves were affected, showing yellowing or necrosis in Bhima Super, which subsequently reached 42% during the next sampling after the 10th day of the recovery phase, i.e., 17 days. The pre-infection waterlogging displayed a similar level of affected leaves to that of sole waterlogged plants. On the other hand, the pre-infection water logging treatment exhibited ~25% higher damage to the leaves than the sole inoculum-sprayed plants. In the case of Bhima Shakti, the waterlogging treatment showed 31%–35% damage to the leaves, while *C. gloeosporioides*-treated plants showed only 15% damage to the leaves. After 7 days of secondary treatment, both post-infection and pre-infection water logging displayed similar levels of damaged leaves to that of sole water-logged plants, i.e., ~39%–44%. On the other hand, the sole inoculum-sprayed plants showed ~24% of damage, which was ~8% higher than the control plants. This indicates that waterlogging has a prominent effect in determining leaf health (**Figure 3**).

### Effect on photosynthetic pigments

**Figure 4:**
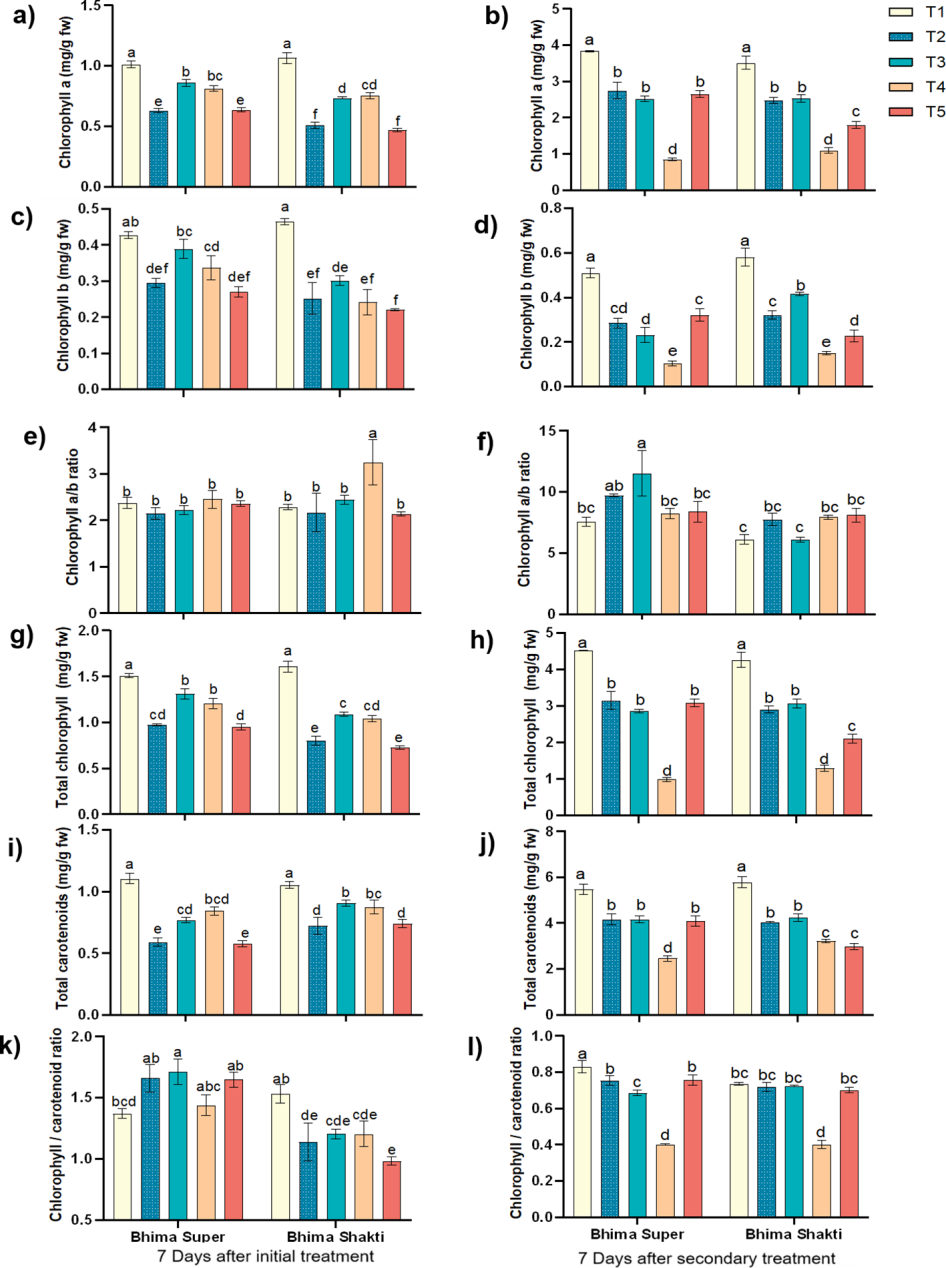
Effect of waterlogging and anthracnose on photosynthetic pigments; chlorophyll a **(a, b)**, chlorophyll b **(c, d)**, chlorophyll a/b **(e, f)**, total chlorophyll **(g, h)**, total carotenoids **(i, j)** and chlorophyll/carotenoid **(k, l)** ratio in Bhima Super and Bhima Shakti after 7 days of initial treatment **(a, c, e, g, i, k)** and 7 days of secondary treatment **(b, d, f, h, l)**. T1: Control, T2: Waterlogging, T3: Anthracnose, T4: Post- infection waterlogging, T5: Pre- infection waterlogging. The mean from the three biological replicates is represented along with standard error and Duncan’s multiple comparison for mean separation.

The reduction in chlorophyll *a* content was highest in waterlogged conditions across cultivars after 7 days of initial treatment. In Bhima Super, the reduction in chlorophyll *a* content was ~37%, while in Bhima Shakti it was ~52%. The *Colletotrichum* sp. inoculum-sprayed plants showed a 14%–19% reduction in chlorophyll content in Bhima Super, while a 29%–31% reduction was observed in Bhima Shakti. The post-infection water logging treatment displayed 3.5 - to 4.5-fold lower chlorophyll *a* content than the control, which is ~2.3- to 2.9- fold lower than the sole inoculum-sprayed plants. At the same time, the other treatment showed a similar level of chlorophyll *a*, i.e., 2.4 – 2.5 mg/g fw, which was ~1.3- to 1. 9-fold lower than control plants (**Figures 4a, b**). Chlorophyll *b* exhibited a similar trend to chlorophyll *a* across varieties and treatments at 7 days of initial treatment of water logging and inoculum spray. Similarly, the post-infection water logging markedly reduced the chlorophyll *b* content, i.e., 2.22- to 2. 77-fold lower than sole inoculum-treated plants. In Bhima Super, the pre-infection water logging exhibited a similar level of chlorophyll *b* content to that of sole waterlogged plants, i.e., ~0.28–0.32 mg/g fw, while in Bhima Shakti post-infection water logging showed a lower chlorophyll *b* content than sole waterlogged or sole inoculum-treated plants (**Figures 4c, d**). After 7 days of initial treatment, the total chlorophyll level was reduced by ~46% in Bhima Super and ~31% in Bhima Shakti in water-logged conditions, and with inoculum spray, the reduction was ~26% and ~16% in Bhima Super and Bhima Shakti, respectively, in comparison to control plants. In Bhima Super, the total chlorophyll level was substantially reduced by post-infection waterlogging, which is nearly threefold lower, while pre-infection waterlogging showed a similar level of total chlorophyll content to that of other treatments. In Bhima Shakti, both pre- and post-infection waterlogging markedly reduced the total chlorophyll content, with post-infection waterlogging exhibiting a higher level of reduction (**Figures 4g, h**). Total carotenoids also displayed a similar trend to that of total chlorophyll. After 7 days of initial treatment, the reduction in carotenoid level was 46%–47% in Bhima Super and 29%–31% in Bhima Shakti under waterlogging conditions. While the *Colletotrichum* sp.- inoculum-sprayed plant exhibited 23%–30% reduction in Bhima Super and 12%–16% in Bhima Shakti. The post-infection waterlogging exhibited 40.76% reduction in total carotenoid level in Bhima Super, while Bhima Shakti displayed 24.10% reduction (**Figures 4i, j**).

It was observed that the chlorophyll *a*/*b* ratio was unaltered at 7 days of initial treatment across cultivars and across treatments. Post-infection and pre-infection waterlogging did not show any significant difference in chlorophyll *a*/*b* ratio in Bhima Super in comparison to control plants, while in Bhima Shakti, the chlorophyll *a*/*b* ratio displayed a similar level to that of sole waterlogged plants (**Figures 4e, f**). Enhancement in the ratio of chlorophyll to carotenoid was observed in Bhima Super after 7 days of initial treatment, while in Bhima Shakti the ratio was significantly reduced in both treatments. The post-infection waterlogging exhibited a ~40% reduction in the ratio in both varieties (**Figures 4k, l**).

### Effect on the stress indicators

**Figure 5:**
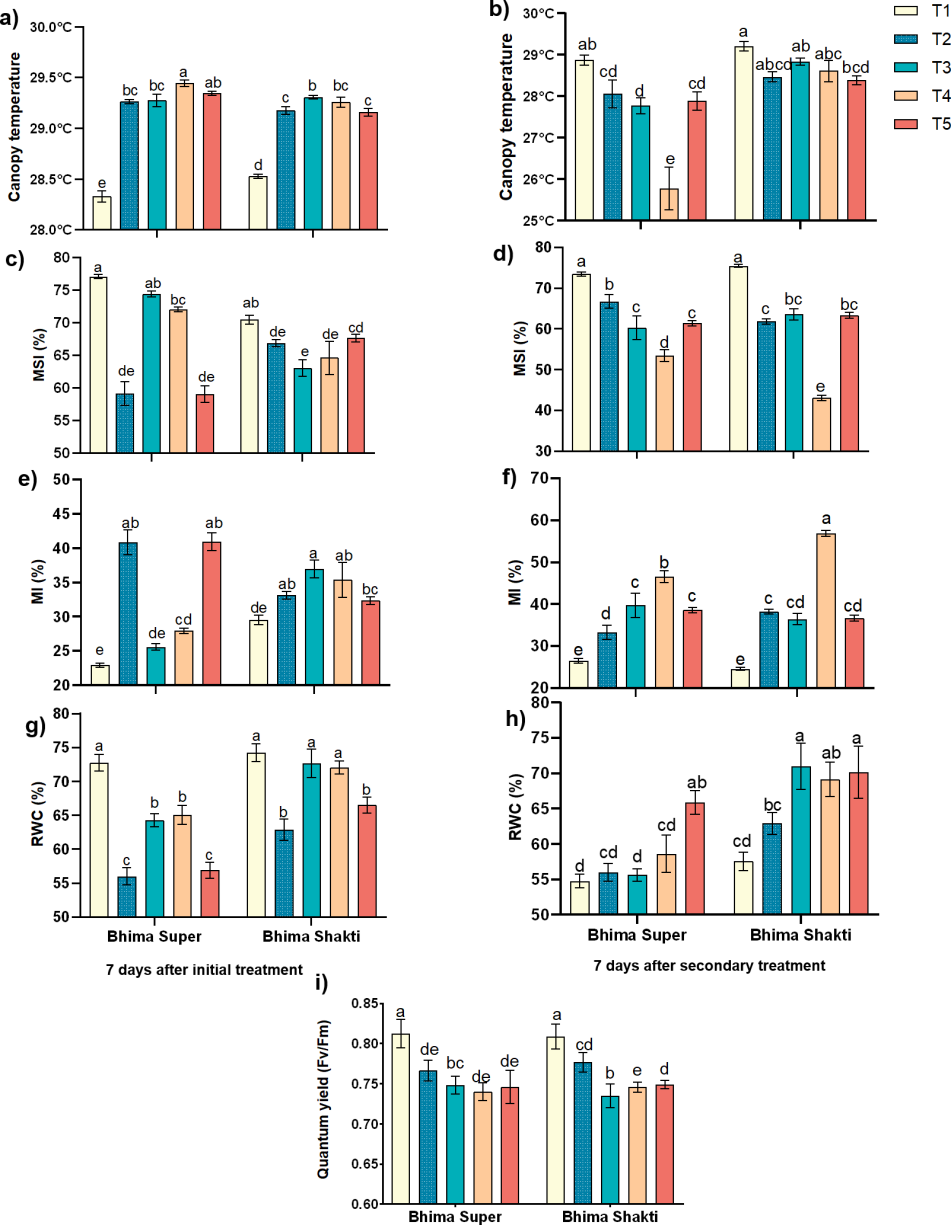
Effect of waterlogging and anthracnose on stress indicators; Canopy temperature **(a, b)**, membrane stability index (MSI) **(c, d)**, membrane injury (MI) **(e, f)**, relative water content (RWC) **(g, h)**, quantum yield **(i)** in Bhima Super and Bhima Shakti after 7 days of initial treatment **(a, c, e, g)** and 7 days of secondary treatment **(b, d, f, h, i)**. T1: Control, T2: Waterlogging, T3: Anthracnose, T4: Post- infection waterlogging, T5: Pre- infection waterlogging. The mean from the three biological replicates is represented along with standard error and Duncan’s multiple comparison for mean separation.

The waterlogging and the *Colletotrichum* sp. inoculum significantly altered the stress indicators: canopy temperature, membrane stability index, injury to the membrane, relative water content, and quantum yield of PS II. The canopy temperature is mainly determined by the changes in stomatal conductance. The plants exposed to stress show a reduction in stomatal conductance, and subsequently the temperature of the leaf surfaces increases. In our experiment, after 7 days of initial stress, the canopy temperature was increased by 0.9 °C to 1 °C across both cultivars and both treatments, suggesting the closure of stomata to conserve water and inhibit fungal entry into the plants. However, after the subsequent second treatment, the canopy temperature was reduced across all of the treatments, with post-infection waterlogging in Bhima Super showing a marked reduction in temperature (~26°C). The initial waterlogged plants showed a reduction in temperature during the recovery phase, indicating an increase in stomatal conductance during the recovery phase (**Figures 5a, b**).

Membrane stability is determined by estimating the membrane stability index (MSI) and membrane injury (MI). After 7 days of initial treatment, the MSI was significantly affected by waterlogging in Bhima Super, exhibiting 59% MSI, which is ~23% lower than control plants, while the pathogen-treated plants showed a lower effect on MSI. In Bhima Shakti, the inoculum spray has more negative effects than waterlogging, i.e., ~10% reduction. Furthermore, the post-infection waterlogging showed a significant negative effect on MSI, with Bhima Super showing 11% and Bhima Shakti displaying 32.27% reduction in MSI compared to sole inoculum-treated plants. In Bhima Super, pre-infection waterlogging inhibited the plants’ recovery. Bhima Super exhibited 40% membrane injury in waterlogged conditions, while Bhima Shakti displayed 35% membrane injury in *C. gloeosporioides* inoculum. Post-infection water logging enhanced the membrane injury by 17% in Bhima Super and 56.3% in Bhima Shakti (**Figures 5c–f**). Similarly, the notable decrease in RWC was also observed (**Figures 5g, h**).

Quantum yield determines the damage caused to PS II. The damage to the PS II was at a similar level across treatments after the secondary treatment across cultivars, except in waterlogging recovered plants of Bhima Shakti, which had a lesser reduction in quantum yield than the other treatments, indicating the recovery level of the plant, while this recovery was suppressed by *C. gloeoporioides* (**Figure 5i**).

### Effect on the level of protein and antioxidant enzyme activity

**Figure 6:**
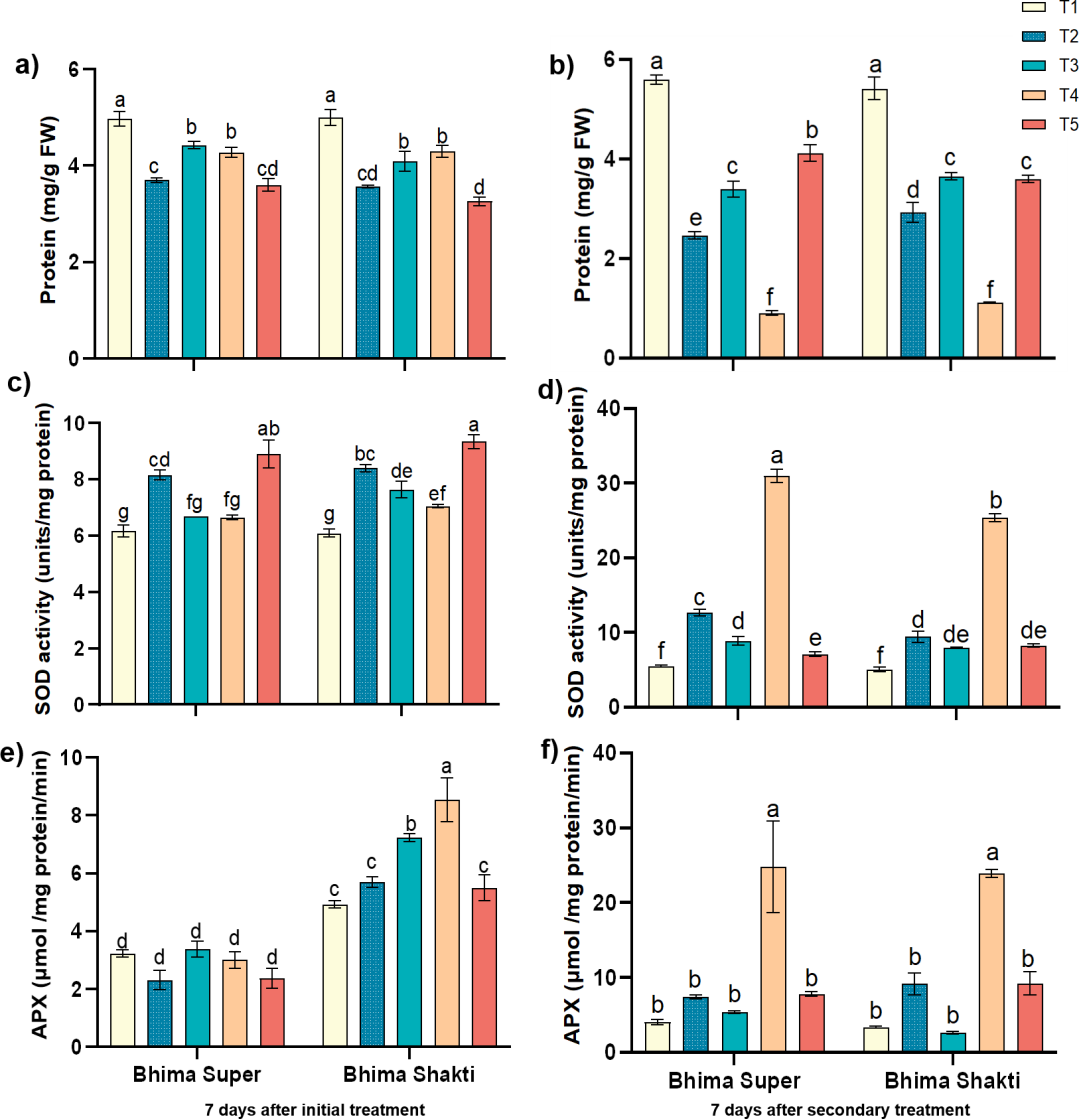
Effect of waterlogging and anthracnose on protein **(a, b)**, antioxidants super oxide dismutase activity (SOD) **(c, d)** and ascorbate peroxidase activity (APX) **(e, f)**, in Bhima Super and Bhima Shakti after 7 days of initial treatment **(a, c, e)** and 7 days of secondary treatment **(b, d, f)**. T1: Control, T2: Waterlogging, T3: Anthracnose, T4: Post- infection waterlogging, T5: Pre- infection waterlogging. The mean from the three biological replicates is represented along with standard error and Duncan’s multiple comparison for mean separation.

The protein level in the leaves of the onion plants varied between 3.2 and 4.9 mg/g FW after 7 days of initial treatment across cultivars and across treatments. The protein level reduction was highest under waterlogged conditions in both cultivars (25%–30%), which subsequently reached to 2.4 – 2.9 mg/g FW, while *C. gloeosporioides*-inoculated plants displayed only 10%–18% reduction in protein level, which reached 4.1 – 4.2 mg/g FW from 4.9 mg/g FW. After secondary treatment, post-infection waterlogging has substantially reduced the protein level by 69%–73% in comparison to sole *C. gloeosporioides* inoculum-treated plants (**Figures 6a, b**). When we estimated the activity of superoxide dismutase (SOD), we observed that waterlogging augmented the SOD activity by 24%–34% with the activity of 8.2 – 8.9 units/mg protein in Bhima Super and 8.4 – 8.9 units/mg protein in Bhima Shakti, while the *C. gloeosporioides* inoculum augmented the SOD activity only in Bhima Shakti (12%–15%). The secondary treatment of post-infection waterlogging has significantly enhanced the activity of SOD by 68%–71% in both cultivars, while pre-infection waterlogging has curtailed the SOD activity in comparison to sole waterlogging recovered plants (**Figures 6c, d**). After 7 days of initial treatment, the ascorbate peroxidase activity was found to be curtailed by waterlogging by 26% in Bhima Super and while enhanced in Bhima Shakti by 13%, which subsequently enhanced during a recovery phase. On the other hand, the APX activity was significantly enhanced by anthracnose inoculum in Bhima Shakti by 25%–30%. This augmentation was curtailed subsequently. Post-infection waterlogging also displayed a significant enhancement in the activity of APX, which is fourfold to ninefold higher than the sole *C*. *gloeosporiodes* inoculum spray, while pre-infection waterlogging displayed a similar level of APX activity to that of the waterlogging recovered plant (**Figures 6e, f**).

## Discussion

Waterlogging causes substantial yield losses in onion, reaching up to 50%–70% during the Indian monsoon season (Samra et al., 2006). The combination of cloudy conditions, drizzling rains, and high humidity further supports the establishment of anthracnose caused by *Colletotrichum gloeosporioides*. Considering these factors, the present study examined the impact of waterlogging on the development of anthracnose disease in onions and compared the effects of pre− and post−infection waterlogging on the severity of this disease complex.

Plants exposed to waterlogging display diverse adaptive strategies (Pan et al., 2021). In our study, waterlogging significantly reduced the plant height across cultivars. Interestingly, *C. gloeosporioides*-inoculated plants exhibited greater plant height compared to the control, mainly due to pseudostem elongation. This elongation response was also seen under combined stress, where pre−infection waterlogging suppressed elongation, while the elongation triggered by the pathogen was maintained under post−infection waterlogging. Similar reductions in plant height under waterlogging stress (5%–22% across genotypes) have also been reported previously (Gedam et al., 2022). In rice, adaptive responses to submergence involve the Sub1A gene, which suppresses the ethylene pathway and gibberellin (GA) biosynthesis while enhancing brassinosteroid (BR) production, leading to restricted internode elongation as part of a quiescence strategy (Zhang et al., 2025). Whether onions possess a comparable regulatory mechanism remains unknown; however, our results suggest that a similar regulatory process might contribute to height reduction under waterlogging, which requires further investigation. The neck elongation observed with *C. gloeosporioides* inoculation in this study is consistent with previous findings (Dutta et al., 2024). Secondary infections by *Gibberella moniliformis* are known to stimulate GA synthesis, resulting in abnormal neck elongation in onion (Alberto and Aquino, 2010). Based on these observations, we hypothesize that pathogen−induced neck elongation in onion could involve hormonal changes, particularly GA and ethylene signaling, although this was not directly tested in the present study and should be considered a potential mechanism rather than a confirmed pathway.

Photosynthetic pigments serve as important components for plant photosynthesis, and alterations in pigment content and composition directly impact the photosynthetic rate. In our study, waterlogging emerged as a major stress factor, causing a significant reduction in photosynthetic pigments compared to pathogen inoculation alone. However, the plants showed the ability to recover their pigment levels once the waterlogging stress was alleviated. Notably, when the pathogen was introduced after the recovery from waterlogging, it did not significantly impact the photosynthetic pigment levels. In contrast, when waterlogging was imposed following pathogen inoculation, the reduction in photosynthetic pigments was significantly greater than that observed under either stress individually. Ghodke et al. (2018) also reported a significant reduction in the chlorophyll content of onion plants exposed to waterlogging. The available literature survey did not show any work related to photosynthetic pigment in onion in relation to *C. gloeosporiodes* infection. However, Chakraborty et al. (2019) has shown that infection with *C. gloeosporioides* was able to significantly curtail the photosynthetic pigment when infected to common bean (*Phaseolus vulgaris* L.). As the content of these photosynthetic pigments decreased, there was a corresponding reduction in photosynthetic capacity. We observed a reduction in plant height, chlorophyll *a*, chlorophyll *b*, total chlorophyll, and total carotenoid content under waterlogged conditions across cultivars. Further post-infection waterlogging has substantially reduced the photosynthetic pigments. Under waterlogged conditions, the reduction in stomatal conductance, the degradation of chlorophyll, the onset of leaf senescence, and the yellowing of leaves diminish the light-absorbing capacity of the leaves, consequently causing a decrease in the photosynthetic rate and plant growth (Kuai et al., 2014; Yan et al., 2018).

Reactive oxygen species (ROS) are a natural by-product of plant cell metabolism. Hypoxic conditions can result in enhanced intracellular ROS during waterlogging stress (Pucciariello et al., 2012). Specifically, superoxide radicals (·O_2_), hydroxyl radicals (·OH), and hydrogen peroxide (H_2_O_2_) exhibit potent oxidizing activity, leading to lipid peroxidation and damage to membranes, proteins, and DNA through oxidation and severe impairment of cell membranes and organelles (Sharma et al., 2012; Baxter et al., 2014). In our study, we found a significant decline in protein level under waterlogged conditions. Though inoculum load showed a reduction in protein level, but it was not as significant as that of the waterlogged plant. Furthermore, regarding the inoculum load contained in the plant when exposed to waterlogging (post-infection waterlogging), the decline in protein level was more severe than individual stress or pre-infection waterlogging. Similarly, membrane damage was also higher in post-infection waterlogging. Our results aligned with those of Li et al. (2025) who also showed that the protein content in both the leaves and roots of Welsh onion decreases progressively with an increase in the duration of waterlogging. R T Alberto (2014) noted that the levels of protein, reducing sugars, total sugars, and phenols were lower in all *C. gloeosporioides*-infected plants compared to the uninoculated controls. Furthermore, the activities of antioxidant enzymes, specifically superoxide dismutase (SOD) and ascorbate peroxidase (APX), were elevated under waterlogged conditions compared to the control and pathogen-inoculated plants. Among the interaction treatments, plants subjected to waterlogging following pathogen inoculation (post-infection waterlogging) exhibited a significantly higher antioxidant enzyme activity than those exposed to waterlogging prior to inoculation (pre-infection waterlogging). These findings suggest that waterlogging acts as a primary stressor, markedly influencing plant physiological and metabolic responses. Plants respond to waterlogging stress by upregulating both enzymatic and non-enzymatic antioxidant systems to confer tolerance, a response also reported by Dubey et al. (2021) in onion. It is noteworthy that apart from damaging the cells, the ROS can also serve as a signaling molecule during stress conditions. The signal transduction is mainly mediated by the pivotal enzyme NADPH oxidase, which is involved in ROS generation. It has been shown that in *Arabidopsis*, *Atrboh* D gene expression was upregulated by waterlogging, which subsequently leads to a higher production of ROS, including hydrogen peroxide (H_2_O_2_). This further induces the upregulation of the *ADH1* gene, thereby enhancing ethanol fermentation and improving the survival rates of the plants (Sun et al., 2018).

**Figure 7:**
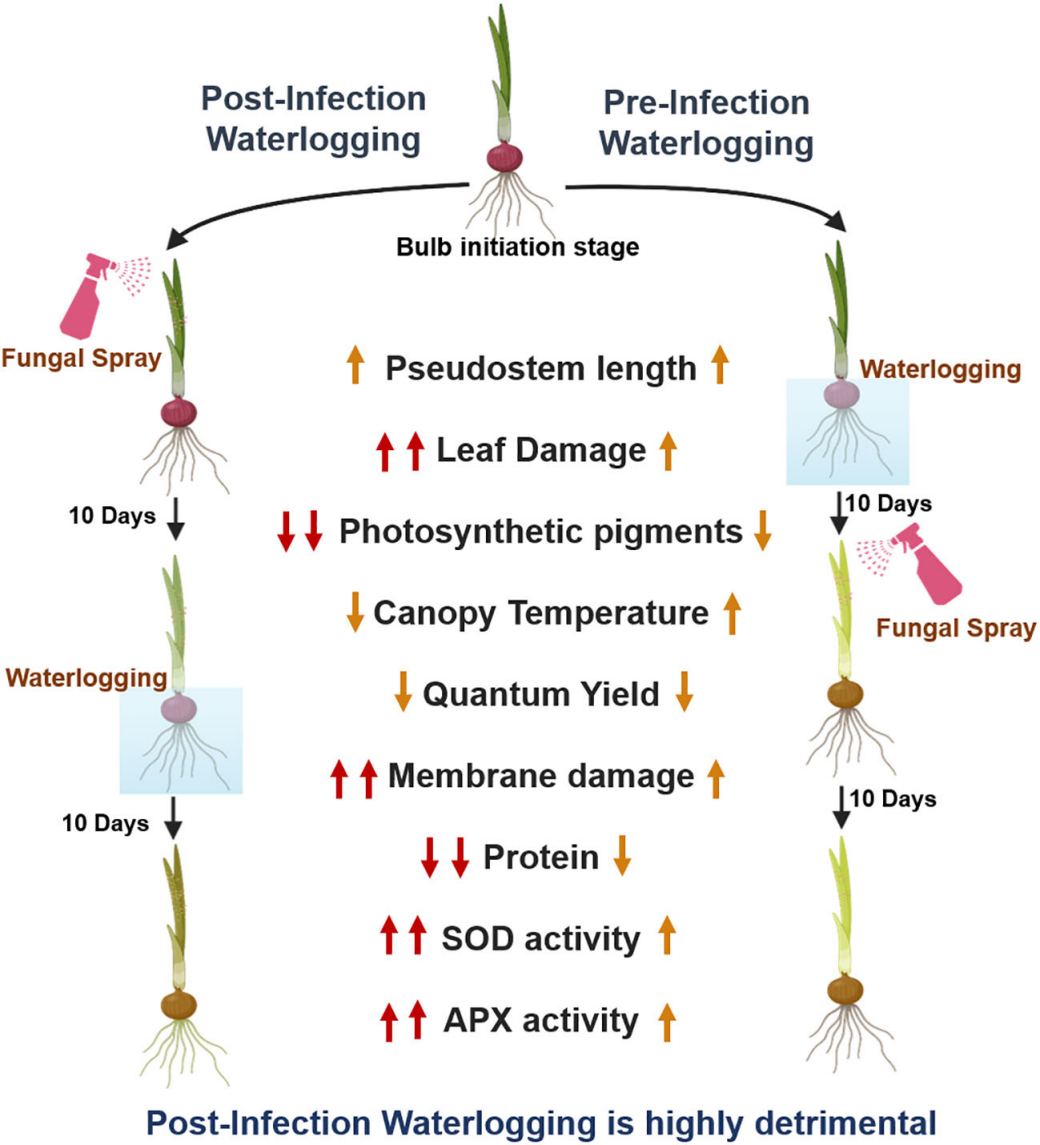
Model depicting the effect of post-infection waterlogging and pre-infection waterlogging on morpho-physio-biochemical parameters of the onion plants.

Several studies have shown that waterlogging and flooding alter the plant microbiome, which significantly impacts their disease resistance. The moist and humid microclimate favors the germination and proliferation of disease—causing significant yield loss (Savary et al., 2019). However, in contrast to this general understanding, our study highlighted that the impact of the pathogen depends on the timing of the waterlogging. The waterlogging prior to the infection limited the establishment of *C. gloeosporioides*, while post-infection waterlogging has significantly enhanced it. This contrasting response might have arisen due to the hypoxic stress created by waterlogging, which may have enhanced the plant’s defense response as a result of activation of the general stress response (Chung and Lee, 2020). Supporting this, Hsu and Shih (2013) also showed that waterlogging/flooding activates the WRKY transcription factor, which regulates the expression of immune-responsive genes which confer resistance to microbial pathogens. It is plausible that a similar immune signature molecule might be involved in onion, whose activation is determined by the time of onset of waterlogging. This study highlights the novel interactive study of waterlogging and airborne pathogen infection in onion and highlights the pivotal role of the timing of waterlogging, which determines the disease progress. We found that post-infection waterlogging is more destructive, which has significantly altered the morpho-physio-biochemical parameters of onion (**Figure 7**). Further advanced transcriptomic and metabolomic studies can provide clear insights into disease suppression or progression under waterlogging.

## Data Availability

The original contributions presented in the study are included in the article/**Supplementary Material**. Further inquiries can be directed to the corresponding authors.
