# Supplementary material for: Deciphering the complex interplay of waterlogging and anthracnose twister disease in onion (Allium cepa L.)
Source: Front Plant Sci. 2025 Sep 19;16:1580269. doi: 10.3389/fpls.2025.1580269 (PMC12494068; doi:10.3389/fpls.2025.1580269)
Supplement: Supplementary file 1 [file DataSheet1.pdf]

Supplementary Table 1: Weather data at experimental site during *Kharif/monsoon*, 2023

Year	Month	Temperature (°C)		Relative Humidity (%)		Cumulative Rainfall (mm)	Rainy days
		Max	Min	Max	Min		
2023	June	34.90	22.30	81.00	45.10	65	5
	July	30.31	22.18	88.65	63.35	28	7
	August	30.84	21.06	86.48	58.55	14	4
	September	30.85	21.61	88.90	61.80	219.8	10
	October	32.65	19.46	83.81	36.81	32.2	2

(Source: Meteorology department of NIASM, Baramati, Pune)
